# Characterization and phylogenetic analysis of Krüppel-like transcription factor (KLF) gene family in tree shrews (*Tupaia belangeri chinensis*)

**DOI:** 10.18632/oncotarget.13883

**Published:** 2016-12-10

**Authors:** Ming Shao, Guang-Zhe Ge, Wen-Jing Liu, Ji Xiao, Hou-Jun Xia, Yu Fan, Feng Zhao, Bao-Li He, Ceshi Chen

**Affiliations:** ^1^ Key Laboratory of Animal Models and Human Disease Mechanisms of Chinese Academy of Sciences and Yunnan Province, Kunming Institute of Zoology, Chinese Academy of Sciences, Kunming, Yunnan, China; ^2^ Faculty of Life Science and Technology, Kunming University of Science and Technology, Kunming, Yunnan, China; ^3^ Department of Laboratory Animal Science, Kunming Medical University, Kunming, Yunnan 650500, China; ^4^ Medical Faculty, Kunming University of Science and Technology, Kunming, Yunnan, China

**Keywords:** KLF, tree shrew, expression, motif, KLF5

## Abstract

Krüppel-like factors (KLFs) are a family of zinc finger transcription factors regulating embryonic development and diseases. The phylogenetics of KLFs has not been studied in tree shrews, an animal lineage with a closer relationship to primates than rodents. Here, we identified 17 KLFs from Chinese tree shrew (*Tupaia belangeri chinensis*). KLF proteins are highly conserved among humans, monkeys, rats, mice and tree shrews compared to zebrafish and chickens. The CtBP binding site, Sin3A binding site and nuclear localization signals are largely conserved between tree shrews and human beings. Tupaia belangeri (Tb) KLF5 contains several conserved post-transcriptional modification motifs. Moreover, the mRNA and protein expression patterns of multiple tbKLFs are tissue-specific. TbKLF5, like hKLF5, significantly promotes NIH3T3 cell proliferation *in vitro*. These results provide insight for future studies regarding the structure and function of the tbKLF gene family.

## INTRODUCTION

Human Krüppel-like factors (hKLFs) belong to a transcription factor family with 17 members containing three highly conserved zinc finger DNA binding domains at their C-terminus [1, 2]. KLFs play important roles in a diverse array of physiological and pathological cellular processes, including cell proliferation, apoptosis, migration and differentiation [3, 4]. KLFs promote the pluripotent state of embryonic stem cells [5, 6], and regulate embryonic and system development, including the cardiovascular [7], respiratory [8], digestive [9], hematological [10], skeleton [11], nervous and immune systems [12, 13]. Moreover, KLFs participate in tissue formation and remodeling, such as cardiac remodeling [14, 15], wound healing [16], placenta development [17], angiogenesis [18, 19] and gluconeogenesis [20]. Finally, the alteration of KLFs is involved in a number of diseases, such as cancers [21], cardiovascular diseases [22] and metabolic disorders [23].

The C-terminus of all KLFs contains three tandem C2H2 zinc finger domains that directly bind to GC-rich sequences, including GC and GT boxes (also known as the CACCC box) in target gene promoters [24]. The three zinc fingers have the consensus sequence: “CX4CX12HX3HX7CX4CX7DX4HX3HX7CX2CX12 HX3H” (Xn: separation of n residues, C: cysteine, H: histidine) [2]. Most KLFs contain classical nuclear localization signals (NLS) in their zinc finger (ZF) domains. hKLF1 has two NLSs: NLS1 (275–296) and NLS2 (293–376) [25]. Similarly, hKLF4 and 13 have one NLS within the ZF domain and another one adjacent to the first ZF domain [26, 27]. However, hKLF8 has two non-classical NLSs; one is in the first two ZF domains in the C-terminus, and the other is located in the 151–200 amino acid region within the regulatory N-terminus [28].

Although the KLF gene family has a highly conserved C-terminal DNA binding domain, each KLF has a distinct function in cellular processes because of great variation in its N-terminal sequence that can interact with different transcriptional activators or repressors. For example, KLF3, 5, 8 and 12 harbor the CtBP binding site PVDLS/T and KLF9, 10, 11, 13, 14 and 16 harbor the Sin3A binding site AA/VXXL [4]. A phylogenetic analysis of 17 hKLFs defined their evolutionary history and structural characteristics [4]. The KLF family members were divided into four groups: (1) KLF3, 8 and 12; (2) KLF1, 2, 4–7; (3) KLF9-11, 13, 14 and 16; and (4) KLF15 and 17 [4]. The KLFs in groups 1 and 3 serve as transcriptional repressors by interacting with the corepressors CtBP or Sin3A, whereas the KLFs in group 2 function as transcriptional activators. Intriguingly, protein interaction motifs were not identified in KLF15 and 17 [4].

KLFs were first identified as an evolutionarily conserved subfamily homologous to *Drosophila melanogaster* Krüppel protein [29]. KLFs have been studied in various species, including humans [1, 30, 31], murine [32], porcine [33], chicken [34], zebrafish [35], Daphnia [36], and *C. elegans* [37], as well as metazoans [38]. However, the KLF gene family has never been reported in tree shrews, a valuable and novel animal model for human diseases [39].

Tree shrews (*Tupaia belangeri*) are widely distributed in Southwest China, Southeast Asia and South Asia and are placed in the order of Scandentia [40]. In recent years, tree shrews have gained increasing attention due to their great potential as an experimental animal model of human diseases [39, 40], such as myopia [41], diabetes [42, 43], depression [44, 45], fatty liver disease [46], viral infection [47–49], hepatocellular carcinoma [50] and breast cancer [51–53]. Whole genome sequencing results demonstrated that the tree shrew lineage has a relatively closer relationship to primates than rodents [54].

Since tree shrews are close to human beings than rodents in evolution, it may serve as a better animal model to study human disease mechanisms [54]. Herein, we elucidated the structural characteristics of 17 KLF factors in tree shrews. This study provides fundamental information on the gene structure and phylogeny of the KLF gene family in the Chinese tree shrews.

## RESULTS

### Characterization of tree shrew KLFs

The tree shrew has a closer relationship to humans than rodents. Because 17 *KLF* genes have been identified in both humans and mice, tree shrews should also have 17 *KLFs*. We obtained 13 tbKLF coding sequences from the NCBI database and the tbKLF6 coding sequence from the tree shrew database. TbKLF2, 5 and 9 genes were cloned using RT-PCR and sequenced. All 17 tbKLF gene accession numbers are listed in Table 1. The coding regions of tbKLFs are 735 to 1500 bp in length and encode 245 to 500 amino acid residues.

**Table 1:** Sequences of Tree shrew KLFs

KLFs	Size CDS/cDNA (bp)	Size protein (aa)	Accessionnumber
KLF1	1083	361	XM_006160997
KLF2	1029	343	KT948683
KLF3	1032	344	XM_006169246
KLF4	1446	482	XM_006150608
KLF5	1374	458	KT948685
KLF6	852	284	TSDBG00002459
KLF7	912	304	XM_006142447
KLF8	945	315	XM_006162614
KLF9	717	239	KT948684
KLF10	1437	479	XM_006145578
KLF11	1500	500	XM_006140828
KLF12	1209	403	XM_006139823
KLF13	744	248	XM_006167284
KLF14	954	318	XM_006158848
KLF15	1230	410	XM_006169767
KLF16	858	286	XM_014585496
KLF17	1161	387	XM_006153770

### Tree shrew KLFs are evolutionarily conserved

We sought to define the amino acid sequence similarity among tree shrew, human and mouse KLFs. The whole amino acid similarities between tree shrew and human KLFs range from 41.87% to 98.51%. TbKLF3-7, 9, 10 and 12 proteins show greater than 90% identity to their human orthologs. However, tbKLF13, 14, 16 and 17 only share < 60% similarity (Table 2). Consistently, the full KLF amino acid sequences identity between human and mouse range from 42.03% to 97.02%. Mouse (m) KLF3, 4, 7, 9, 12 and 13 show high similarities (> 90%), but KLF1, 14 and 17 exhibit low similarities (< 70%). Among these KLFs, mKLF17 protein has the lowest similarity (∼40%), as well as tbKLF13 and 16. Overall, tbKLFs and their human homologs have an average 85.56% and 83.47% identity on full amino acid and nucleotide sequences respectively, which is slightly higher than those (83.26% and 82.3%) between mouse and their human homologs. Thus, both tbKLFs and mKLFs are homologous to their human corresponding genes. This multigene family is evolutionary conserved, although some members, including tbKLF13, 14, 16, 17, seem to be a little divergent during evolution.

**Table 2:** Sequence similarities of KLFs from tree shrew, human and mouse

Human KLF	Tree shrew % homology with human				Mouse % homology with human			
	Zn finger		CDS		Zn finger		CDS	
	Nucleotide	Protein	Nucleotide	Protein	Nucleotide	Protein	Nucleotide	Protein
HsKLF1	90.60	94.74	79	75.55	83.95	90.12	70.06	66.75
HsKLF2	89.56	100	87.07	73.60	85.19	96.30	82.26	87.36
HsKLF3	92.18	100	88.63	97.10	88.07	100	86.71	95.36
HsKLF4	93.83	100	89.60	91.77	90.12	100	86.86	90.12
HsKLF5	93.42	100	95.27	96.50	87.65	96.30	86.58	88.50
HsKLF6	92.59	100	90.85	97.53	95.47	100	78.16	84.59
HsKLF7	97.12	100	94.19	97.69	95.47	100	92.74	97.02
HsKLF8	93.83	98.77	79.81	80.22	93.42	100	85.37	82.73
HsKLF9	95.88	98.77	94.96	96.33	94.65	100	94.15	96.72
HsKLF10	92.18	98.77	89.95	92.29	89.71	98.77	84.13	85.42
HsKLF11	91.77	95.06	83.53	80.44	88.48	97.53	76.78	73.10
HsKLF12	94.65	100	96.86	98.51	87.24	100	90.65	97.01
HsKLF13	91.77	95.06	55.11	41.87	95.47	98.77	88.70	91.84
HsKLF14	95.88	100	63.51	50.61	89.71	96.30	74.23	65.85
HsKLF15	94.65	100	83.13	82.93	90.12	100	83.37	85.10
HsKLF16	90.64	94.74	68.51	42.29	86.42	92.59	81.84	85.99
HsKLF17	82.72	79.01	72.47	56.71	76.54	72.84	56.57	42.03
**Average**	**92.55**	**97.35**	**83.09**	**79.53**	**89.28**	**96.44**	**82.30**	**83.26**

Next, we investigated the phylogenetic relationship of KLFs among different species to track the evolutionary history of tbKLF members. An evolutionary tree was established using the Maximum Likelihood method [56] based on the JTT matrix-based model [59] with KLF amino acid sequences among human, monkey, tree shrew, rat, mouse, chicken and zebrafish (Supplementary Table S2). The bootstrap consensus tree inferred from 1000 replicates is taken to represent the evolutionary history of the taxa analyzed [58].

**Figure 1:**
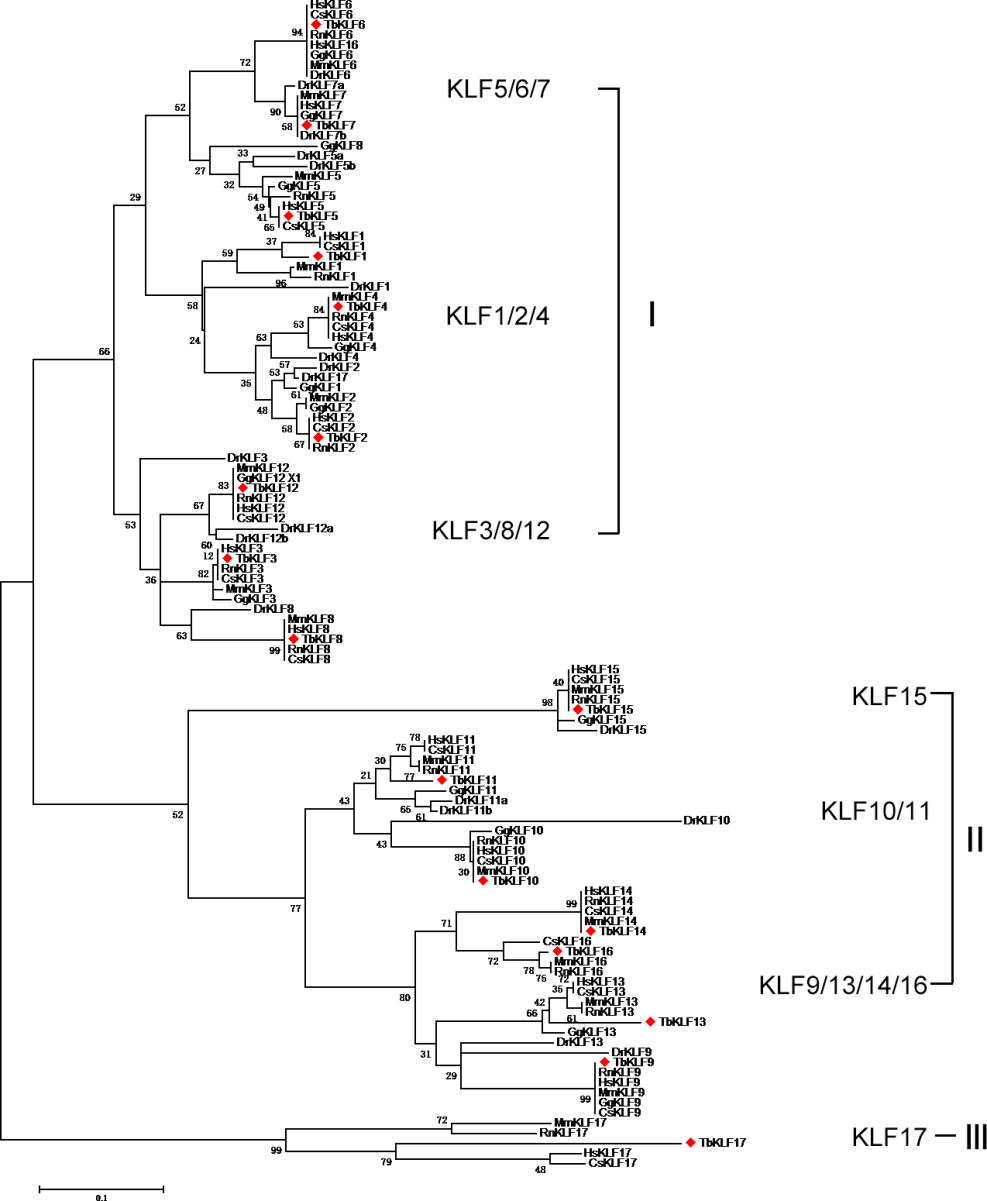
Maximum Likelihood Phylogenetic tree of KLFs from human, monkey, rat, mouse, tree shrew, chicken and zebrafish sequences The evolutionary history was inferred by using the Maximum Likelihood method based on the JTT matrix-based model. The bootstrap consensus tree inferred from 1000 replicates is taken to represent the evolutionary history of the taxa analyzed. Branches corresponding to partitions reproduced in less than 50% bootstrap replicates are collapsed. The percentage of replicate trees in which the associated taxa clustered together in the bootstrap test (1000 replicates) are shown next to the branches. The analysis involved 116 amino acid sequences. All positions containing gaps and missing data were eliminated. Evolutionary analyses were conducted in MEGA5. The species acronyms are Hs, Homo sapiens (human); Dr, Danio rerio (zebrafish); Mm, Mus musculus (mouse); Gg, Gallus gallus (chicken); Tb, Tupaia belangeri chinensis (tree shrew); Chlorocebus sabaeus (green monkey); and Rattus norvegicus (rat).

As the associated taxa clustered together in the booststrap test (1000 replicates) are shown next to the branches. The *KLF* gene family was divided into three large groups, which were further divided into several subgroups (Figure 1). Group I consists of three subgroups, including KLF1/2/4, KLF5/6/7 and KLF3/8/12. Group II contains three subgroups, including KLF15, KLF10/11 and KLF9/13/14/16. Interestingly, Group III contains only one member, KLF17. In total, the phylogenetic tree shows members of each subgroup are developed from the same ancestral gene during evolution. Different members suffer from different evolution patterns, including convergent evolution and divergent evolution.

For each subgroup members, the evolutionary conservation of KLFs in mammalian including human, monkey, rat and mouse and tree shrew is higher compared with chicken and zebrafish. Most of KLFs among human, monkey, tree shrew, rat and mouse are closely located on the tree branches, such as KLF4, 7–10, 14 and 15, demonstrating that these species share closer relationship on KLF evolution. Moreover, tbKLF1, 2, 5, 16 and 17 have closer distance to primates than rodents on the inferring evolutionary tree (Figure 1). These data reveal that tbKLF members are not only evolutionary conserved but also closer to primates than rodent, chicken and zebrafish.

### The ZF domains of KLFs in the tree shrew

**Figure 2:**
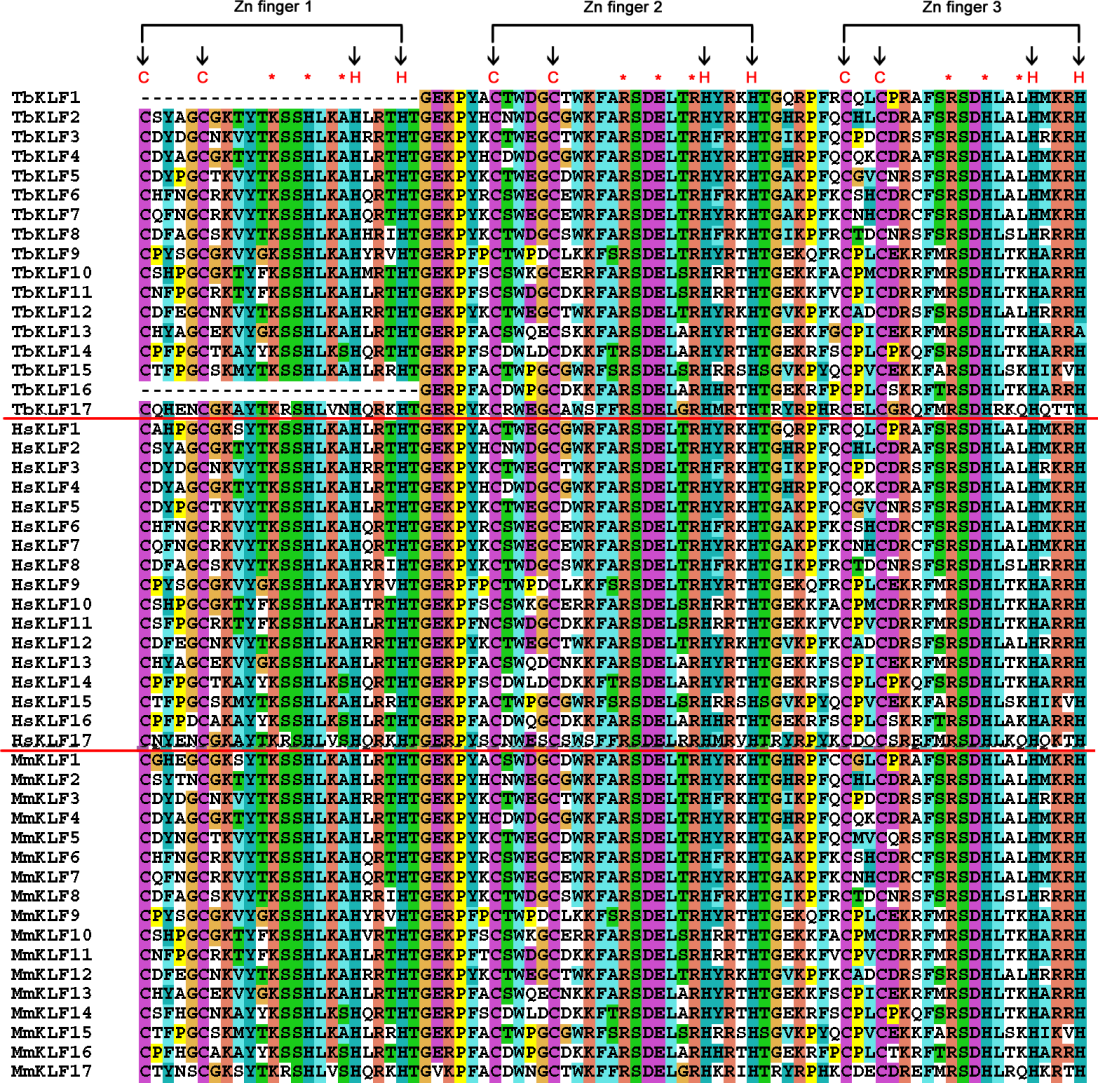
Amino acid residue alignment of KLF zinc finger domains in tree shrews, mice and humans Three zinc fingers are indicated above. The cysteine and histidine in the zinc fingers are indicated by a black arrow, and the DNA binding residues are marked with a red asterisk.

KLF proteins are transcription factors acting as activators and/or suppressors. It seems that all KLFs contain three classical C2H2 ZF domains at their C-terminus, nuclear localization signals (NLS), and transactivation or trans-suppression domains [24, 60]. The ZF domains of KLFs are usually conserved among different species, such as humans and mice (Figure 2). To elucidate whether this conservation is also true for the tree shrew, the ZF domains of 17 tbKLFs were identified (Figure 2). The three C2H2 ZF domains consist of 81 amino acid residues. The first and second ZF domains consist of 23 amino acid residues, while the last domain has only 21 amino acid residues. Intriguingly, tbKLF1 and 16 do not have the first ZF domain. Because of the critical role of the first zinc-finger domain for sequence specific DNA binding, it is a puzzle for tree shrew to lose this key region during evolution. We deduce that the sequences we obtained are isoforms from alternative splicing.

Zinc finger domains are more highly conserved among different species than the entire coding regions due to the variant N-termini. The alignment results showed that the identity of KLF ZF domains between the tree shrew and humans is greater than 90% (even 100%) for all KLFs except KLF17 (Table 2). The average homology of nucleotides and proteins between the tree shrew and humans is 92.55% and 97.35%, respectively. These figures are slightly higher than those between mice and humans. Meanwhile, we built a NJ tree from the zinc finger domains of KLFs among humans, mice and tree shrews (Supplementary Figure S1) with the zinc-finger amino acid sequences collected based on “CX4CX12HX3HX7 CX4CX7DX4HX3HX7CX2CX12HX3H” (Supplementary Table S1). The zinc finger domains of tbKLF1, 2, 5 and 17 located in the tree show a closer evolutionary relationship with their orthologs in humans compared to those in mice. Though the zinc-finger domain of KLF17 formed a divergent branch during evolution, it's still conserved among the three different lineages, such as human, tree shrew and mouse (Supplementary Figure S2).

### A comparative analysis of CtBP and Sin3A binding sites and NLS in tbKLFs

**Figure 3:**
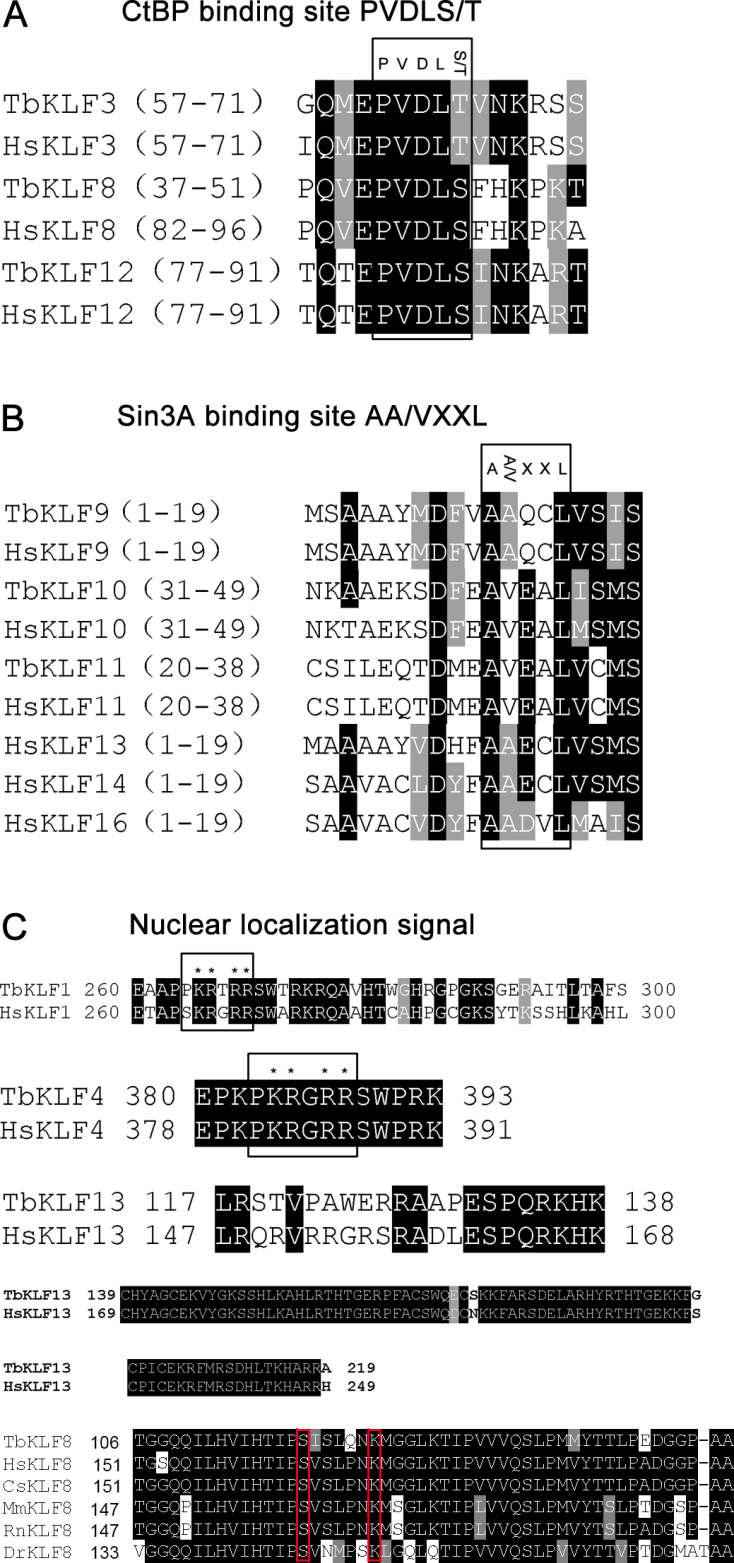
Sequence alignment of conserved CtBP binding motifs, Sin3A binding motifs, and nuclear localization signals in KLFs (**A**) CtBP binding motif PVDLS/T in KLF3, 8 and 12. (**B**) Sin3A binding motif AA/VXXL in KLF9, 10, 11, 13, 14 and 16. (**C**) Nuclear localization signal sequence in KLF1, 4, 5, 8 and 13.

In addition to the highly conserved zinc finger domains at their C-termini, we analyzed the relatively variant N-termini containing several functional motifs, including CtBP binding sites, Sin3A binding sites and NLS. By aligning the functional motifs of tbKLFs to their orthologs in human, we found that tbKLF3, 8, and 12 have a conserved CtBP binding motif PVDLS/T (Figure 3A). Interestingly, PVDLS/T motifs in KLF3 and 12 are localized at the same regions between the tree shrew and humans, while PVDLS/T motifs in KLF8 are localized at different regions in the tree shrew and humans. A conserved Sin3A binding site (AA/VXXL) was identified in hKLF 9-11, 13, 14 and 16. This motif was identified in tbKLF9-11 but not in tbKLF13, 14 and 16 (Figure 3B).

A NLS is essential for transcription factors to localize in the nucleus. We compared the NLS of tbKLFs and hKLFs. hKLF1 contains two NLS. The first one is adjacent to the ZF domain within a highly basic stretch of amino acid residues (Figure 3C). The second one is located within the conserved ZF domain [25]. Sequence alignment results showed that tbKLF1 also has NLS1 and NLS2. Similarly, tbKLF4 and hKLF4 have two NLS that are located at their N-termini and ZF domains [26]. The basic amino acid residues in KLF4 are highly conserved between both species (Figure 3C). In addition, two identified NLS in hKLF13 [27] were identified in tbKLF13 (Figure 3C). Moreover, hKLF8 has been reported to have two non-classical NLS [28]; one is located within a region from 151 to 200 amino acid residues and contains two critical amino acid residues, S165 and K171. Consistently, a motif alignment of KLF8 among tree shrew, human, monkey, rat, mouse and zebrafish sequences show that all species have nearly the same NLS sequence in the N-terminus, especially the two highly conserved residues (Figure 3C). Not surprisingly, other NLS of tbKLFs in the ZF domain are conserved as all ZF domains are conserved between tree shrews and humans.

### The post-translational modification motifs in TbKLF5

The tree shrew is an alternative animal model for breast cancer research because it develops spontaneous breast tumors [61]. Thus, we induced breast tumors in tree shrews using different carcinogens, such as DMBA plus MPA and the PyMT oncogene [52]. Our previous work showed that KLF5 is highly expressed in ERa-PR-HER2 triple-negative breast cancer [63, 64]. KLF5 promotes human breast cancer cell proliferation, survival and migration [65]. The human KLF5 protein undergoes different post-translational modifications, including ubiquitination, phosphorylation, acetylation and sumoylation. We analyzed the conservation of modification sites or motifs responsible for recruiting modification enzymes.

**Figure 4:**
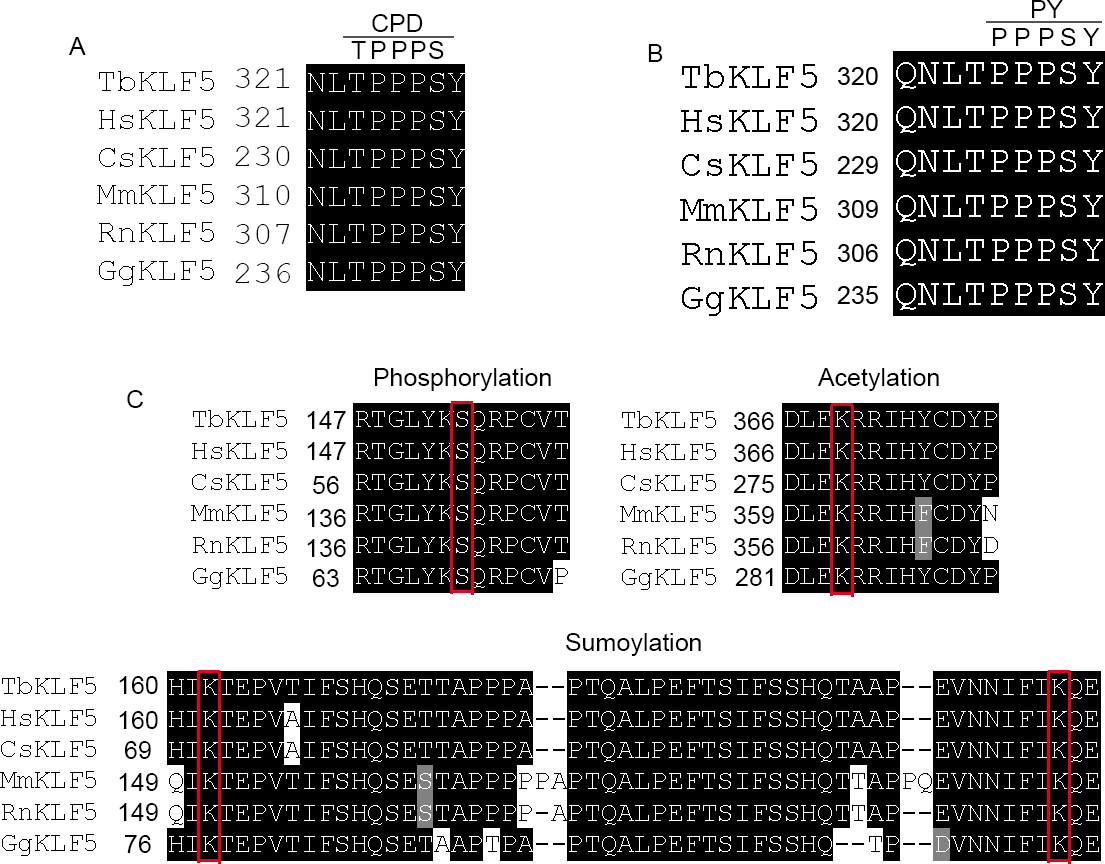
Post-translational modification sequences are conserved in tbKLF5 (**A**) The Fbw7 binding CPD motif sequence is conserved among tree shrew, human, monkey, mouse, rat and chicken sequences. (**B**) The WWP1 binding PY motif sequence is conserved among tree shrew, human, monkey, mouse, rat and chicken sequences. (**C**) The PKC phosphorylation site S153, the acetylation site K369 and two sumoylation sites K162 and K209 are conserved in tree shrew KLF5.

Based on the KLF5 CDS phylogenetic tree, tbKLF5 is closer to human and monkey sequences than mouse, rat and chicken sequences (Supplementary Figure S2). Motif alignments showed that tbKLF5 has the conserved CPD (Figure 4A) and PY motifs (Figure 4B) in its transactivation domain. These motifs are responsible for recruiting Fbw7 and WWP1 E3 ubiquitin ligases [66, 67]. These conserved CPD and PY motifs indicate that the tbKLF5 protein may be regulated by Fbw7 and WWP1 through the ubiquitin-proteasome pathway. In addition, the PKC phosphorylation site S153 [68], the p300 acetylating site K369 [69], and two sumoylation sites K162 and K209 [70] are conserved among tree shrew, human, monkey, mouse, rat and chicken proteins (Figure 4C).

### The expression patterns of tbKLFs

KLFs play important roles in cell stemness, proliferation, survival, migration, and differentiation. Abnormal expression of KLF proteins in different human and mouse tissues can trigger several diseases, including cancers, cardiovascular diseases and metabolic diseases [4]. We sought to determine the relative mRNA and protein expression levels of several KLFs in different tree shrew tissues.

**Figure 5:**
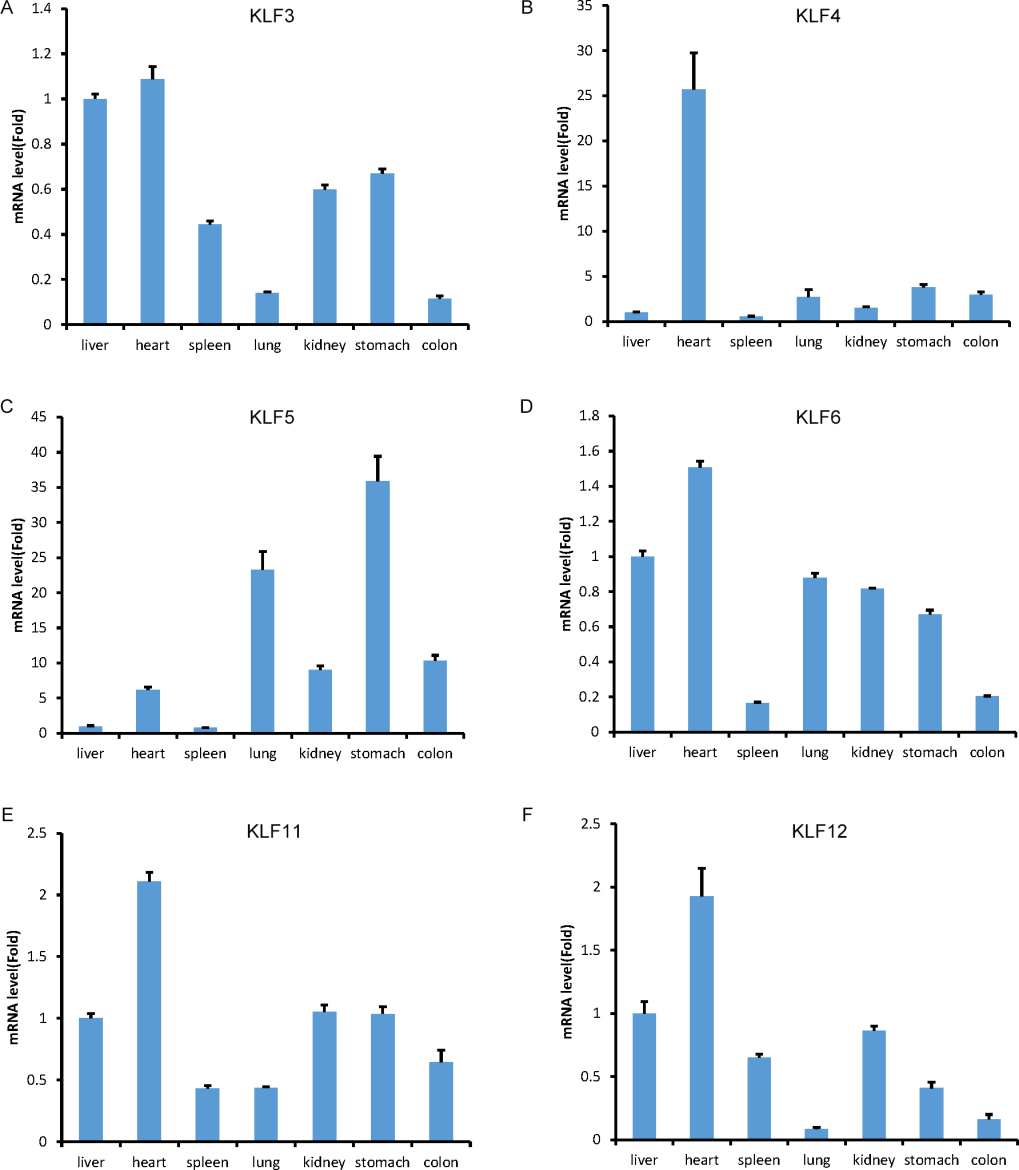
The mRNA expression levels of tree shrew KLF3, 4, 5, 6, 11 and 12 in the liver, heart, spleen, lung, kidney, stomach and colon tissues, as measured by RT-qPCR The expression level of KLFs in the liver was defined as 1.

We chose six tbKLF genes, including KLF3, 4, 5, 6, 11 and 12, to examine their mRNA levels in the liver, heart, spleen, lung, kidney, stomach and colon using RT-qPCR. Compared to the liver, all of the detected factors have a high mRNA level in the heart (Figure 5). TbKLF3 is relatively highly expressed in the heart, liver, kidney and stomach but lowly expressed in the spleen, lung and colon (Figure 5A). TbKLF4 is just highly expressed in the heart (Figure 5B). TbKLF5 shows high expression levels in the stomach, lung, colon, heart and kidney (Figure 5C). In addition, tbKLF6 is highly expressed in the heart and lowly expressed in the spleen and colon (Figure 5D). TbKLF11 is highly expressed in the heart but lowly expressed in the spleen and lung (Figure 5E). TbKLF12 is highly expressed in the heart and lowly expressed in the lung and colon (Figure 5F). Overall, these tbKLFs are ubiquitously expressed in different tissues, while some factors show tissue-specific expression patterns.

**Figure 6:**
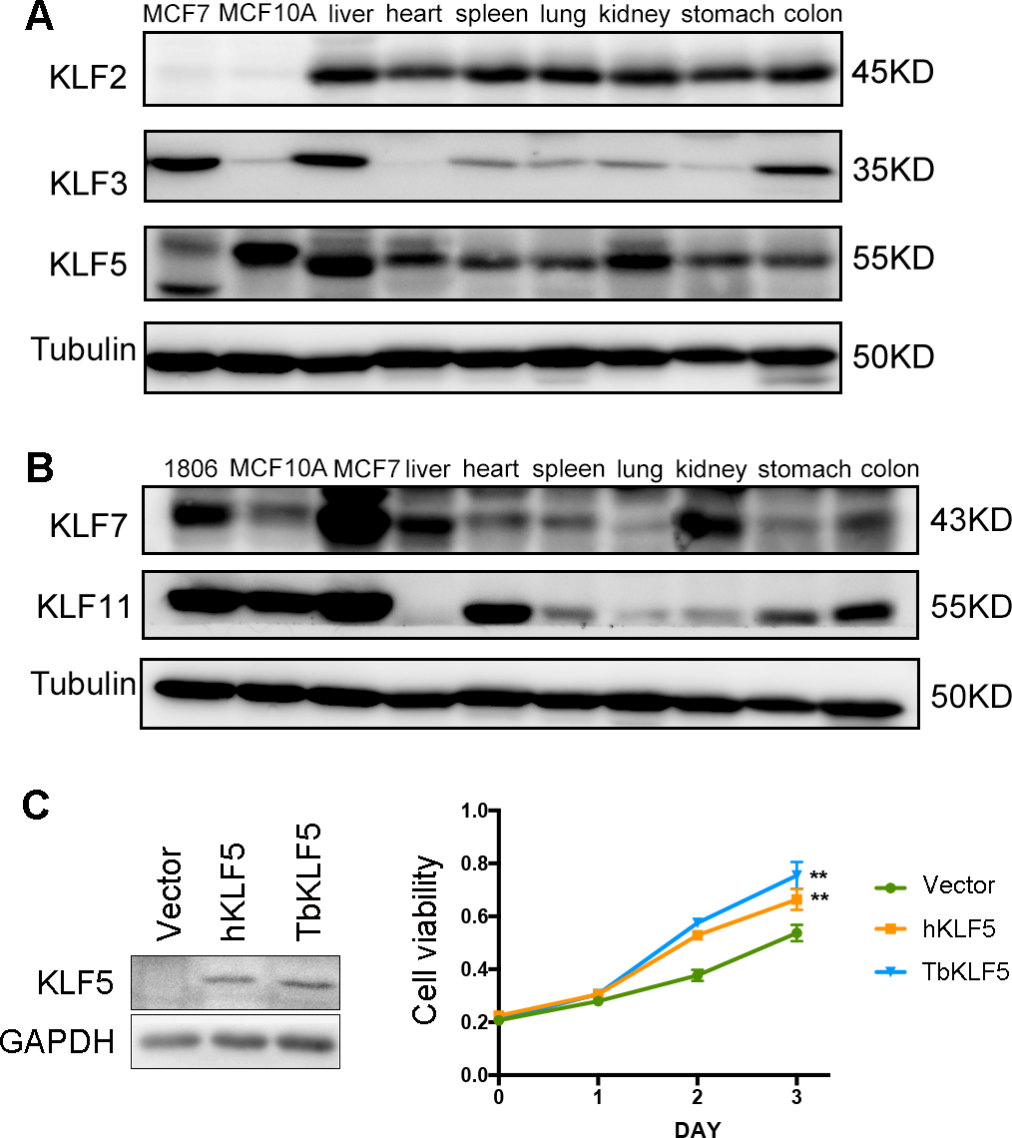
The protein expression levels of tree shrew KLF2, 3, 5, 7 and 11 in different tissues and tbKLF5 promotes cell proliferation like hKLF5 (**A**) The protein expression levels of tbKLF2, 3, and 5 in different tissues was measured by Western blotting. Tubulin was used as the loading control. Two human breast epithelial cell lines, MCF7 and MCF10A, were used as controls. (**B**) The protein expression levels of tbKLF7 and 11 in different tissues was measured by Western blotting. Three human breast epithelial cell lines, HCC1806, MCF10A and MCF7, were used as controls. (**C**) Overexpression of TbKLF5 or hKLF5 in NIH3T3 cells promote cell proliferation. The expression of TbKLF5 or hKLF5 in NIH3T3 cells was measured by Western blotting (Left panel). Overexpression of TbKLF5 or hKLF5 promote NIH3T3 cell proliferation in the course of three days, as measured by the SRB assay (Right panel). **p < 0.01.

Then, we examined the protein levels of tbKLF2, 3, 5, 7 and 11 in the liver, heart, spleen, lung, kidney, stomach and colon by Western blotting based on the availability of antibodies (Figure 6). KLF2 is ubiquitously expressed in all tested tissues (Figure 6A). The KLF3 protein level is highly detected in the liver and colon but almost undetectable in the heart (Figure 6A). Both KLF5 and 7 are expressed at high levels in the liver and kidney (Figure 6). High protein expression levels of KLF11 were detected in the heart and colon (Figure 6B). Thus, each tbKLF shows a unique expression pattern at both the mRNA and protein levels. They are extensively expressed in various tissues but each member shows a tissue-specific expression pattern. Notably, compared to the corresponding mRNA levels of tbKLF3, 5 and 11, the protein expression pattern is not always consistent with the mRNA expression pattern.

### TbKLF5 promotes cell proliferation like hKLF5

To compare the functions of tbKLFs and hKLFs, we took KLF5 as an example and compared the pro-proliferation function of tbKLF5 and hKLF5 in NIH3T3 cells. KLF5 has been shown to promote NIH3T3 cell proliferation *in vitro* [71]. As shown in Figure 6C, when tbKLF5 and hKLF5 were overexpressed in NIH3T3 cells, both tbKLF5 and hKLF5 significantly and equally promote cell proliferation compared to the vector control. These results suggest that tbKLF5 functions similarly to hKLF5 in terms of promoting cell proliferation. In another word, the pro-proliferation function of KLF5 is conserved between tree shrews and human beings.

## DISCUSSION

KLF transcription factors belong to a large zinc finger domain family. In mammals, 17 KLF members have been implicated in many physiological and pathological processes [4]. Elucidating the structures, functions and expression patterns of the KLF members is important for understanding their essential roles in normal tissue development and diseases. Considering that tree shrews are becoming novel disease animal models, it is important to understand the biology of tree shrews at the molecular level. However, KLFs in the tree shrew have not been investigated previously.

In this study, we first identified 17 tbKLF protein sequences and defined their evolutionary status compared to other species. Then, we analyzed the conservation of their functional domains, including the ZF domain, CtBP binding site, Sin3A binding site and NLS. In addition, we analyzed the conservation of known KLF5 post-translational motifs or sites in the tree shrew. Finally, we examined the mRNA and protein expression patterns of several KLFs in different tree shrew tissues.

A phylogenetic analysis showed that *KLF* genes in mammals could be divided into two large groups [1]. In porcine, two groups were further divided into three subgroups according to their clusters in the phylogenetic tree [33]. In addition to porcine, similar KLF subgroups were also classified in humans [31] and mice [32]. As expected, tbKLFs were also divided into the same subgroups according to the phylogenetic analysis, indicating that they might also be functionally conserved among human, mouse, porcine and tree shrew sequences. A phylogenetic tree using tree shrew, human, monkey, rat, mouse, chicken and zebrafish sequences demonstrate that the tbKLF family likely originates from the same ancestor. Though most mammalian KLFs show a similar evolutionary pattern, some tbKLFs, such as KLF1, 11, 13, 16 and 17, have unique evolution pathways. They obviously separate from both primates and rodents on the evolutionary branches. In particular, KLF17 was a distinct member that is distantly related to the other KLFs.

KLF transcription factors contain highly conserved DNA binding domains at their C-termini and variant N-termini, which interact with cofactors to activate and/or repress gene transcription. We identified 3 conserved C2H2 ZF domains [30] from all the tbKLFs except tbKLF1 and tbKLF6. It's weird that tbKLF1 and 6 don't have the first ZF domain. This may be caused by mRNA alternative splicing. A phylogenetic tree indicated that the ZF domains are highly conserved among human, mouse and tree shrew proteins. In the N-termini, the CtBP or Sin3A binding motifs and NLS are also largely conserved. These conserved sequence regions may be essential for their functions.

KLF5 is an important transcription factor associated with many human cancers, such as breast cancer [64, 65, 72], gastric cancer [73], colorectal cancer [74], bladder cancer [75] and prostate cancer [76]. Post-translational modifications are vital for regulating KLF5 activity. The alignment results showed that tbKLF5 has conserved CPD and PY motifs for recruiting E3 ubiquitin ligases [3, 63]. Furthermore, tbKLF5 and hKLF5 have the same S153 and S303 sites for phosphorylation, K369 site for acetylation and K162 and K209 site for sumoylation. Importantly, both tbKLF5 and hKLF5 promote NIH3T3 cell proliferation similarly. These results suggest that the tree shrew may serve as an alternative animal model for human diseases related to KLF5. In addition to tbKLF5, it is worth exploring other tbKLF post-transcriptional modifications that are related to many important human diseases.

The expression levels of KLFs in different tissues may indicate their functions in organ development, homeostasis and diseases. In the tree shrew, we determined the relative mRNA and protein levels of several KLFs in the adult liver, heart, spleen, lung, kidney, stomach and colon. The high expression levels of *KLF5* mRNA in the lung and colon are consistent with that in humans [77]. *TbKLF4* is highly expressed in the heart and modestly expressed in the lung and colon at the mRNA level. However, *mKLF4* is not highly expressed in the heart, although it is highly expressed in the lung and colon [78]. Thus, *KLF4* may play a role in the maintenance of tree shrew, but not mouse, cardiac function.

The protein levels of tbKLF3, 5 and 11 are not consistent with their mRNA levels in the same tissues. Post-translational modifications may contribute to tbKLF protein expression. Using the KLF5 protein as an example, phosphorylation, acetylation, ubiquitination, and sumoylation are known to change protein levels without affecting mRNA levels [3, 4].

In summary, we identified 17 KLF members in the tree shrew and performed a comprehensive phylogenetic analysis. The high phylogenetic conservation in the sequences of tree shrew, human, mouse and other species indicates the functional significance of tbKLFs. Finally, we demonstrated that several tbKLFs are widely expressed in different adult tissues. Our study provides insight for further functional study of the *KLF* gene family in tree shrews.

## MATERIALS AND METHODS

### Identification of tbKLF gene sequences

The genome sequence of the tree shrew was completed by second-generation sequencing technology described previously [54]. The coding sequences of KLF6 were obtained directly from the tree shrew database (http://www.treeshrewdb.org/), which was built and supported by the Kunming Institute of Zoology, Chinese Academy of Science [55]. The rest of complete coding sequences of tbKLF1, 3, 4, 7, 8, 10–16 and 17 were retrieved from the NCBI database (http://www.ncbi.nlm.nih.gov). Because tbKLF2, 5, 9 have neither been identified nor predicted, we cloned and identified the CDSs of tbKLF2, 5, 9.

The F2 outbreed Chinese tree shrews were fed in the animal center of Kunming Institute of Zoology, Chinese Academy of Science. The liver, heart, spleen, lung, kidney, stomach and colon were harvested from a six-month-old adult female tree shrew using a tissue homogenizer (TIANGEN, OSE-Y20). Total RNA was extracted from different tissues using TRIzol (Life Technologies, Massachusetts, USA). cDNAs were generated using the Revert Aid First Strand cDNA Synthesis Kit (Thermo Scientific, Massachusetts, USA). cDNAs from the lung and liver were used as PCR templates, and PCR reactions were conducted using standard reaction conditions. The PCR primers for tsKLF2, 5, and 9 were designed according to their homologous sequences from human, mouse and pig. The PCR primers were as follows: tbKLF2-F: 5′-CGTCCT TCTCCACTTTCGCC-3′; tbKLF2-R: 5′-CAGGTGGTCA GAGCGTGAG-3′; tbKLF5-F: 5′-ATGGCTACGCGGGT GTTGACCATGAGCGCCCGCCTGGG-3′; tbKLF5-R: 5′-TCAGTTCTGGTGCCTCTTCAT-3′; tbKLF9-F: 5′-AT GTCCGCGGCCGCCTACATGGACT-3′; and tbKLF9-R: 5′-TCACAAAGGGTTGGCCAGCGCCTTT-3′. The target genes were cloned into the pGEM-T vector (Promega, Cat#A3600, Shanghai) and sequenced. The sequences were aligned to their human and mouse homologs and then deposited into GenBank (accession numbers: KT948683, KT948685 and KT948684). All GenBank accession numbers of the 17 tbKLFs are listed in Table 1.

### Comparative and phylogenetic analysis

The KLF coding sequences from various species, including human, monkey, rat, mouse, zebrafish, and chicken, were obtained from GenBank (accession numbers are listed in Supplementary Table S2). Multiple and pairwise alignment of the whole amino acid sequences among or between KLF orthologs were conducted using the Clustal X2.1 program. After comparative analysis, Some conserved domains,including the ZF domain, CtBP binding, Sin3A binding domain and NLS, were then marked by the BoxShade (http://www.ch.embnet.org/software/BOX_form.html).

The phylogenetic analysis was performed by MEGA5 (http://www.megasoftware.net) using the Maximum Likelihood (ML) method [56] and Neighbor-Joining (NJ) [57] method with bootstrapping analyses [58]. In brief, we firstly made complement alignment of the whole amino acid sequences of KLF family among seven lineages, including human, monkey, tree shrew, rat, mouse, chicken and zebrafish and then eliminated mismatch sequences and gaps at both ends. This data containing 116 amino acid sequences was used to reconstruct phylogenetic tree by both ML and NJ methods on MEGA5, with the bootstrap test value setting as 1000.

### RT-qPCR

Real-time qPCR was used to examine the expression patterns of *tbKLFs* at the RNA level. cDNAs from the seven tissues were used as templates. SYBR Select Master Mix (ABI, Cat#4472908, Carlsbad) was used for qPCR in an ABI-7900 HT. The cycling condition was 95°C for 2 min, 95°C for 15 s, and 60°C for 1 min for 40 cycles. The ΔΔCt method was used to analyze *KLF* mRNA levels, and b-actin was used as the loading control. Primers for qPCR were designed to skip exons to avoid genomic DNA contamination. The primer sequences were as follows:

KLF3 F: 5′- GCTCCCATTTGAAAGCACAC -3′;

R: 5′- TTCGTCAGACCGAGCAAAC -3′;

KLF4 F: 5′- GCGAGTCTGACATGGCTGT -3′;

R: 5′- CTCCGTTCTCCAGGTCTGTG -3′;

KLF5 F: 5′- CGCATCCACTACTGCGATTA -3′;

R: 5′- TGAGTCCTCAGGTGAGCTTTT -3′;

KLF6 F: 5′- AAAGCTCCCACTTGAAAGCA -3′;

R: 5′- ACTTCTTGCAAAACGCCACT -3′;

KLF11 F: 5′- TTCCCACCTTAAGGCTCATC -3′;

R: 5′- AACTTCTTCTCCCCTGTGTGAG -3′;

KLF12 F: 5′- ACTATTGTCGTGCCGCTTTT -3′;

R: 5′- AAGGTCACATTTGGCAGGTC -3′;

Actin F: 5′- CATCCGTAAGGACCTGTATG -3′; and

R: 5′- GTAACAGTCCGCCTAGAAGC -3′.

### Western blotting

Fresh tissues of the liver, heart, spleen, lung, kidney, stomach and colon were harvested in lysis buffer (50 mM Tris-HCl (pH 7.4), 150 mM NaCl, 1 mM EDTA, 1% Triton X-100) with 1% protease inhibitor cocktail (Sigma P8340, Shanghai). The Bio-Rad Dc Protein Assay (BIO-RAD, Hercules) was used to quantify protein concentration. The following antibodies were used: KLF2 (ABNOVA, Cat#H00010365-A01, Taipei), KLF3 (Abcam, Cat#ab49221-100, Cambridge), KLF5 (homemade), KLF7 (Abcam, Cat#ab58306-100, Cambridge) and KLF11 (Abcam, Cat#ab65029-100, Cambridge) and Tubulin (Sigma, Cat#T5168, Shanghai). All these antibodies have been validated for WB. All experimental protocols were approved by Kunming Institute of Zoology, Chinese Academy of Sciences, animal welfare and ethical committee.

### Cell culture

NIH3T3 was purchased from the American Type Culture Collection (ATCC). NIH3T3 cells were cultured in DMEM containing 10% fetal bovine serum at 37°C in humidified incubator with 5% CO_2_.

### TbKLF5 and hKLF5 overexpression plasmids

TbKLF5 cDNA was amplified by PCR using tree shrew uterus cDNA as a template. The primer sequences are:

Forward 5′-CGGGATCCATGGCTACGCGGGTGTTG ACCATGAGCGCCCGCCTGGG-3′

Backward 5′-CGGAATTCTCAGTTCTGGTGCCTCTT CAT-3′. The PCR products were subcloned into the pBabe-puro retroviral vector by using Bam HI and Eco RI restriction enzyme cleavage sites. The cloned TbKLF5 was confirmed by sequencing. hKLF5 was previously cloned into the same vector.

### Cell proliferation assays

The Sulforhodamine B (SRB, Sigma, St. Louis, MO) assays were performed to measure cell proliferation. NIH3T3 cells were transfected with expression plasmids of hKLF5, TbKLF5, or empty vector. The expression of KLF5 was confirmed by Western blotting. Forty-eight hours after transfection, 5,000 NIH3T3 cells were seeded in 48-well plates in triplicate. The cells were fixed with 200 ml of 10% trichloroacetic acid (TCA, Sigma, St. Louis, MO) for 60 min and then washed 5 times with deionized water and air dried. The cells were stained with 100 ml of 0.4% (W/V) SRB in acetic acid for 5 min. Then the plates were washed 5 times with 1% acetic acid and dried. Following that, 150 ml of 10 mM Tris base was added to each well. Finally, optical densities were measured on an automated spectrophotometric plate reader at a single wavelength of 530 nm. The results are presented as the means ± the standard deviations of three independent experiments. Student's tests were used to compare pairs of variables. Results were considered statistically significant at the level of *p < 0.05 or **p < 0.01.

## SUPPLEMENTARY MATERIALS AND FIGURES




