# Supplementary material for: Characterization and phylogenetic analysis of Krüppel-like transcription factor (KLF) gene family in tree shrews (Tupaia belangeri chinensis)
Source: Oncotarget. 2016 Dec 10;8(10):16325–39. doi: 10.18632/oncotarget.13883 (PMC5369966; doi:10.18632/oncotarget.13883)
Supplement: Supplementary file 1 [file oncotarget-08-16325-s001.pdf]

Characterization and phylogenetic analysis of Krüppel-like transcription factor (KLF) gene family in tree shrews (*Tupaia belangeri chinensis*)

Supplementary Materials

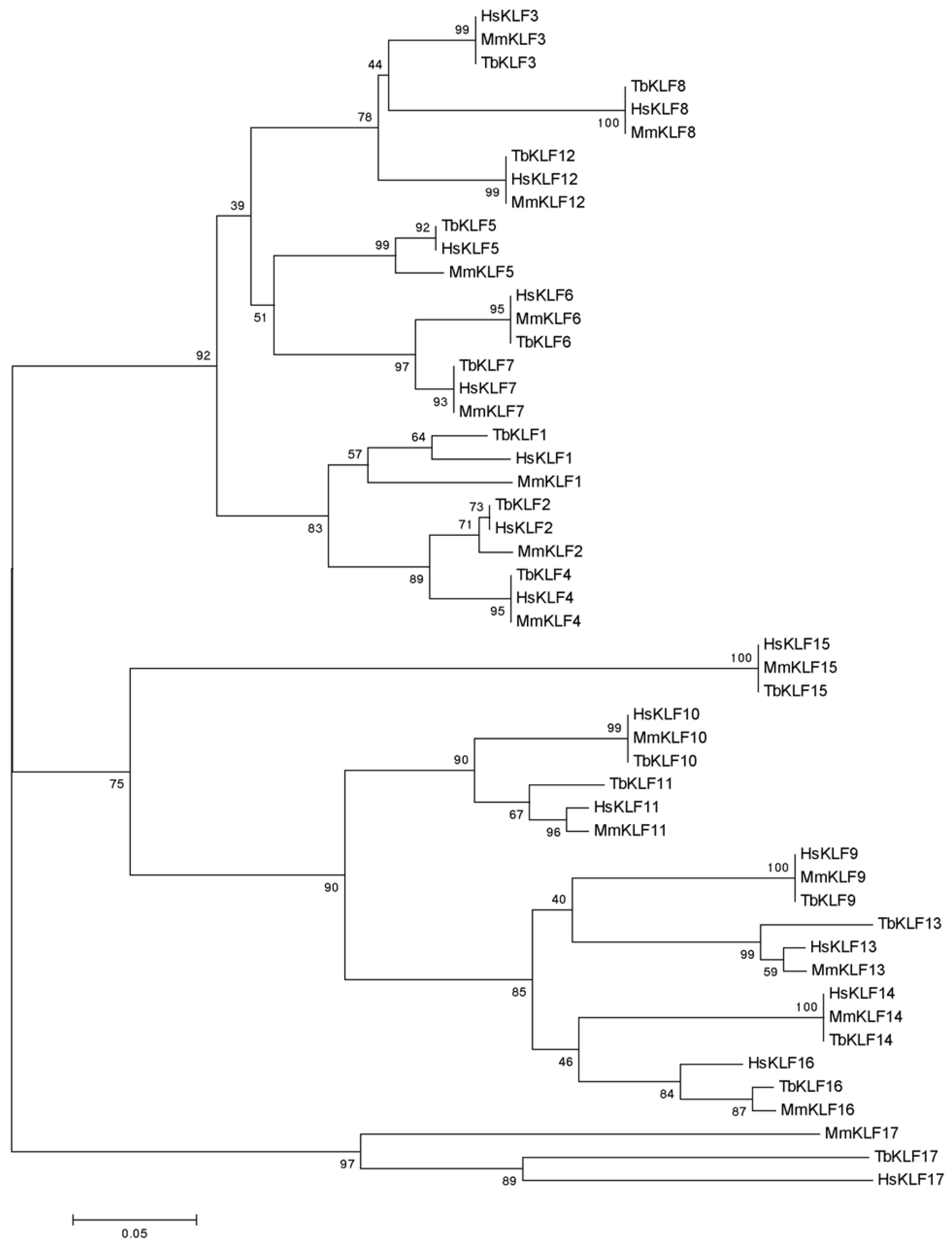

Supplementary Figure S1: A phylogenetic tree of KLF zinc finger domains from human, mouse and tree shrew sequences.

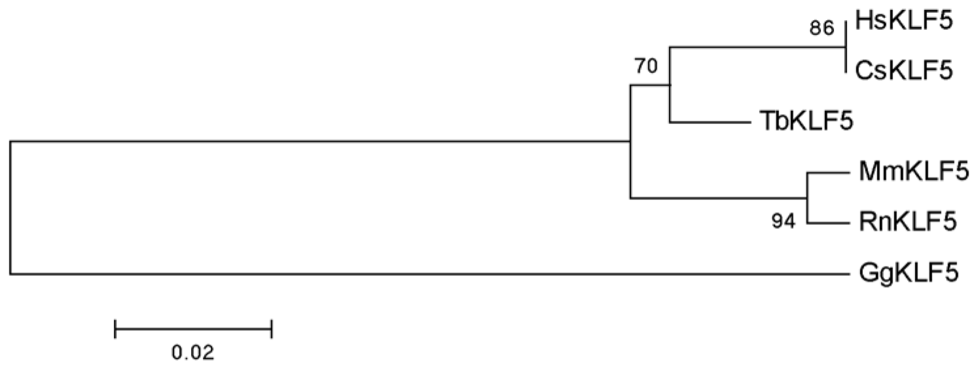

Supplementary Figure S2: A phylogenetic tree of thw KLF5 protein from human, mouse, tree shrew, rat, monkey and chicken sequences.

Supplementary Table S1: The amino acid residue sequences of Zinc-finger domains of KLFs. See Supplementary_Table_S1

Supplementary Table S2: The species source, accession number of KLFs included in our analyses. See Supplementary_Table_S2
