# Supplementary material for: Characterization and phylogenetic analysis of Krüppel-like transcription factor (KLF) gene family in tree shrews (Tupaia belangeri chinensis)
Source: Oncotarget. 2016 Dec 10;8(10):16325–39. doi: 10.18632/oncotarget.13883 (PMC5369966; doi:10.18632/oncotarget.13883)
Supplement: Supplementary file 2 [file oncotarget-08-16325-s002.pdf]

Supplementary Table 1 The amino acid residue sequences of Zinc-finger domains of KLFs

ZF domain	Sequences
**TbKLF1**	GEKPYACTWDGCTWKFARSDELTRHYRKHTGQRPFRCQLCPRAFSRSDHLALHMKRH
**TbKLF2**	CSYAGCGKTYTKSSHLKAHLRTHTGEKPYHCNWDGCGWKFARSDELTRHYRKHTGHR<br>PFQCHLCDRAFSRSDHLALHMKRH
**TbKLF3**	CDYDGCNKVYTKSSHLKAHRRTHTGEKPYKCTWEGCTWKFARSDELTRHFRKHTGIKP<br>FQCPDCDRSFSRSDHLALHRKRH
**TbKLF4**	CDYAGCGKTYTKSSHLKAHLRTHTGEKPYHCDWDGCGWKFARSDELTRHYRKHTGHR<br>PFQCQKCDRAFSRSDHLALHMKRH
**TbKLF5**	CDYPGCTKVYTKSSHLKAHLRTHTGEKPYKCTWEGCDWRFARSDELTRHYRKHTGAK<br>PFQCGVCNRSFSRSDHLALHMKRH
**TbKLF6**	CHFNGCRKVYTKSSHLKAHQRTHTGEKPYRCSWEGCEWRFARSDELTRHFRKHTGAK<br>PFKCSHCDRCFSRSDHLALHMKRH
**TbKLF7**	CQFNGCRKVYTKSSHLKAHQRTHTGEKPYKCSWEGCEWRFARSDELTRHYRKHTGAK<br>PFKCNHCDRCFSRSDHLALHMKRH
**TbKLF8**	CDFAGCSKVYTKSSHLKAHHRIHTGEKPYKCTWDGCSWKFARSDELTRHFRKHTGIKP<br>FRCTDCNRSFSRSDHLSLHRRRH
**TbKLF9**	CPYSGCGKVYGKSSHLKAHYRVHTGERPFPCTWPDCLKKFSRSDELTRHYRTHTGEK<br>QFRCPLCEKRFMRSDHLTKHARRH
**TbKLF10**	CSHPGCGKTYFKSSHLKAHMRTHTGEKPFSCSWKGCERRFARSDELSRHRRTHTGEK<br>KFACPMCDRRFMRSDHLTKHARRH
**TbKLF11**	CNFPGCRKTYFKSSHLKAHLRTHTGEKPFSCSWDGCDKRFARSDELSRHRRTHTGEK<br>KFVCPICDRRFMRSDHLTKHARRH
**TbKLF12**	CDFEGCNKVYTKSSHLKAHRRTHTGEKPYKCTWEGCTWKFARSDELTRHYRKHTGVK<br>PFKCADCDRSFSRSDHLALHRRRH
**TbKLF13**	CHYAGCEKVYGKSSHLKAHLRTHTGERPFACSWQECSKKFARSDELARHYRTHTGEK<br>KFGCPICEKRFMRSDHLTKHARRA
**TbKLF14**	CPFPGCTKAYYKSSHLKSHQRTHTGERPFSCDWLDCDKKFTRSDELARHYRTHTGEK<br>RFSCPLCPKQFSRSDHLTKHARRH
**TbKLF15**	CTFPGCSKMYTKSSHLKAHLRRHTGEKPFACTWPGCGWRFSRSDELSRHRRSHSGVK<br>PYQCPVCEKKFARSDHLSKHIKVH
**TbKLF16**	GERPFACDWPGCDKKFARSDELARHHRTHTGEKRFPCPLCSKRFTRSDHLTKHARRH
**TbKLF17**	CQHENCGKAYTKRSHLVNHQRKHTGERPYKCRWEGCAWSFFRSDELGRHMRTHTRY<br>RPHRCELCGRQFMRSDHRKQHQTTH
**HsKLF1**	CAHPGCGKSYTKSSHLKAHLRTHTGEKPYACTWEGCGWRFARSDELTRHYRKHTGQR<br>PFRCQLCPRAFSRSDHLALHMKRH
**HsKLF2**	CSYAGCGKTYTKSSHLKAHLRTHTGEKPYHCNWDGCGWKFARSDELTRHYRKHTGHR<br>PFQCHLCDRAFSRSDHLALHMKRH
**HsKLF3**	CDYDGCNKVYTKSSHLKAHRRTHTGEKPYKCTWEGCTWKFARSDELTRHFRKHTGIKP<br>FQCPDCDRSFSRSDHLALHRKRH
**HsKLF4**	CDYAGCGKTYTKSSHLKAHLRTHTGEKPYHCDWDGCGWKFARSDELTRHYRKHTGHR

	PFQCQKCDRAFSRSDHLALHMKRH
**HsKLF5**	CDYPGCTKVYTKSSHLKAHLRTHTGEKPYKCTWEGCDWRFARSDELTRHYRKHTGAK PFQCGVCNRSFSRSDHLALHMKRH
**HsKLF6**	CHFNGCRKVYTKSSHLKAHQRTHTGEKPYRCSWEGCEWRFARSDELTRHFRKHTGAK PFKCSHCDRCFSRSDHLALHMKRH
**HsKLF7**	CQFNGCRKVYTKSSHLKAHQRTHTGEKPYKCSWEGCEWRFARSDELTRHYRKHTGAK PFKCNHCDRCFSRSDHLALHMKRH
**HsKLF8**	CDFAGCSKVYTKSSHLKAHRRIHTGEKPYKCTWDGCSWKFARSDELTRHFRKHTGIKP FRCTDCNRSFSRSDHLSLHRRRH
**HsKLF9**	CPYSGCGKVYGKSSHLKAHYRVHTGERPFPCTWPDCLKKFSRSDELTRHYRTHTGEK QFRCPLCEKRFMRSDHLTKHARRH
**HsKLF10**	CSHPGCGKTYFKSSHLKAHTRTHTGEKPFSCSWKGCERRFARSDELSRHRRTHTGEK KFACPMCDRRFMRSDHLTKHARRH
**HsKLF11**	CSFPGCRKTYFKSSHLKAHLRTHTGEKPFNCSWDGCDKKFARSDELSRHRRTHTGEK KFVCPVCDRRFMRSDHLTKHARRH
**HsKLF12**	CDFEGCNKVYTKSSHLKAHRRTHTGEKPYKCTWEGCTWKFARSDELTRHYRKHTGVK PFKCADCDRSFSRSDHLALHRRRH
**HsKLF13**	CHYAGCEKVYGKSSHLKAHLRTHTGERPFACSWQDCNKKFARSDELARHYRTHTGEK KFSCPICEKRFMRSDHLTKHARRH
**HsKLF14**	CPFPGCTKAYYKSSHLKSHQRTHTGERPFSCDWLDCDKKFTRSDELARHYRTHTGEK RFSCPLCPKQFSRSDHLTKHARRH
**HsKLF15**	CTFPGCSKMYTKSSHLKAHLRRHTGEKPFACTWPGCGWRFSRSDELSRHRRSHSGVK PYQCPVCEKKFARSDHLSKHIKVH
**HsKLF16**	CPFPDCAKAYYKSSHLKSHLRTHTGERPFACDWQGCDKKFARSDELARHHRTHTGEK RFSCPLCSKRFTRSDHLAKHARRH
**HsKLF17**	CNYENCGKAYTKRSHLVSHQRKHTGERPYSCNWESCSWSFFRSDELRRHMRVHTRY RPYKCDQCSREFMRSDHLKQHQKTH
**MmKLF1**	CGHEGCGKSYTKSSHLKAHLRTHTGEKPYACSWDGCDWRFARSDELTRHYRKHTGHR PFCCGLCPRAFSRSDHLALHMKRH
**MmKLF2**	CSYTNCGKTYTKSSHLKAHLRTHTGEKPYHCNWEGCGWKFARSDELTRHYRKHTGHR PFQCHLCDRAFSRSDHLALHMKRH
**MmKLF3**	CDYDGCNKVYTKSSHLKAHRRTHTGEKPYKCTWEGCTWKFARSDELTRHFRKHTGIKP FQCPDCDRSFSRSDHLALHRKRH
**MmKLF4**	CDYAGCGKTYTKSSHLKAHLRTHTGEKPYHCDWDGCGWKFARSDELTRHYRKHTGHR PFQCQKCDRAFSRSDHLALHMKRH
**MmKLF5**	CDYNGCTKVYTKSSHLKAHLRTHTGEKPYKCTWEGCDWRFARSDELTRHYRKHTGAK PFQCMVCQRSFSRSDHLALHMKRH
**MmKLF6**	CHFNGCRKVYTKSSHLKAHQRTHTGEKPYRCSWEGCEWRFARSDELTRHFRKHTGAK PFKCSHCDRCFSRSDHLALHMKRH
**MmKLF7**	CQFNGCRKVYTKSSHLKAHQRTHTGEKPYKCSWEGCEWRFARSDELTRHYRKHTGAK PFKCNHCDRCFSRSDHLALHMKRH
**MmKLF8**	CDFAGCSKVYTKSSHLKAHRRIHTGEKPYKCTWDGCSWKFARSDELTRHFRKHTGIKP FRCTDCNRSFSRSDHLSLHRRRH

**MmKLF9**	CPYSGCGKVYGKSSHLKAHYRVHTGERPFPCTWPDCLKKFSRSDELTRHYRTHTGEK QFRCPLCEKRFMRSDHLTKHARRH
**MmKLF10**	CSHPGCGKTYFKSSHLKAHVRTHTGEKPFSCSWKGCERRFARSDELSRHRRTHTGEK KFACPMCDRRFMRSDHLTKHARRH
**MmKLF11**	CNFPGCRKTYFKSSHLKAHLRTHTGEKPFTCSWDGCDKKFARSDELSRHRRTHTGEKK FVCPVCDRRFMRSDHLTKHARRH
**MmKLF12**	CDFEGCNKVYTKSSHLKAHRRTHTGEKPYKCTWEGCTWKFARSDELTRHYRKHTGVK PFKCADCDRSFSRSDHLALHRRRH
**MmKLF13**	CHYAGCEKVYGKSSHLKAHLRTHTGERPFACSWQECNKKFARSDELARHYRTHTGEK KFSCPICEKRFMRSDHLTKHARRH
**MmKLF14**	CSFHGCNKAYYKSSHLKSHQRTHTGERPFSCDWLDCDKKFTRSDELARHYRTHTGEK RFSCPLCPKQFSRSDHLTKHARRH
**MmKLF15**	CTFPGCSKMYTKSSHLKAHLRRHTGEKPFACTWPGCGWRFSRSDELSRHRRSHSGVK PYQCPVCEKKFARSDHLSKHIKVH
**MmKLF16**	CPFHGCAKAYYKSSHLKSHLRTHTGERPFACDWPGCDKKFARSDELARHHRTHTGEK RFPCPLCTKRFTRSDHLTKHARRH
**MmKLF17**	CTYNSCGKSYTKRSHLVSHQRKHTGVKPFACDWNGCTWKFFRSDELGRHKRIHTRYR PHKCDECDREFMRSDHLRQHKRTH

Supplementary Table 2: The species source, accession number of KLFs included in our analyses.

Species	Proteins	Lengths(aa)	Accession number
**Homo sapiens (human)**			
	HsKLF1	362	NP_006554
	HsKLF2	355	NP_057354
	HsKLF3	345	NP_057615
	HsKLF4	479	NP_004226
	HsKLF5	457	NP_001721
	HsKLF6	283	NP_001291
	HsKLF7	302	NP_003700
	HsKLF8	359	NP_009181
	HsKLF9	244	NP_001197
	HsKLF10	480	NP_005646
	HsKLF11	495	NP_001171189
	HsKLF12	402	NP_009180
	HsKLF13	288	NP_057079
	HsKLF14	323	NP_619638
	HsKLF15	416	NP_054798
	HsKLF16	252	NP_114124
	HsKLF17	389	NP_775755
**Danio rerio (zebrafish)**			
	DrKLF1	365	NP_571011
	DrKLF2	380	NP_571931
	DrKLF3	328	NP_571934
	DrKLF4	396	NP_001106955
	DrKLF5a	429	XP_001344916
	DrKLF5b	344	XP_688525
	DrKLF6	283	NP_958869
	DrKLF7a	286	NP_001018479
	DrKLF7b	295	NP_001038231
	DrKLF8	343	NP_001073472
	DrKLF9	216	NP_001122201
	DrKLF10	227	XP_009290995
	DrKLF11a	459	NP_001038406
	DrKLF11b	458	NP_001071072
	DrKLF12a	373	NP_996945
	DrKLF12b	341	NP_001103335
	DrKLF13	258	NP_001070240
	DrKLF15	442	NP_997911
	DrKLF17	409	NP_571798
**Mus musculus(mouse)**			

	MmKLF1	376	NP_034765
	MmKLF2	354	NP_032478
	MmKLF3	344	NP_032479
	MmKLF4	483	NP_034767
	MmKLF5	446	NP_033899
	MmKLF6	318	NP_035933
	MmKLF7	301	NP_291041
	MmKLF8	355	NP_776141
	MmKLF9	244	NP_034768
	MmKLF10	479	NP_038720
	MmKLF11	502	NP_848134
	MmKLF12	402	NP_034766
	MmKLF13	289	NP_067341
	MmKLF14	325	NP_001128565
	MmKLF15	415	NP_075673
	MmKLF16	251	NP_510962
	MmKLF17	341	NP_083692
**Gallus gallus (chicken)**			
	GgKLF1	406	XP_422416
	GgKLF2	380	XP_418264
	GgKLF3	347	XP_427367
	GgKLF4	458	XP_004949426
	GgKLF5	372	XP_417013
	GgKLF6	283	NP_001026140
	GgKLF7	296	XP_004942700
	GgKLF8	485	XP_004940831
	GgKLF9	235	XP_003643067
	GgKLF10	436	XP_427148
	GgKLF11	530	NP_001006417
	GgKLF12 X1	396	XP_003640604
	GgKLF13	277	XP_425065
	GgKLF15	403	XP_004944662
**Chlorocebus sabaeus (green monkey)**	CsKLF1	363	XP_007993641
	CsKLF2	356	XP_007993830
	CsKLF3	346	XP_008015842
	CsKLF4	480	XP_007966680
	CsKLF5	367	XP_007958763
	CsKLF6	284	XP_008000305
	CsKLF7	231	XP_007964212
	CsKLF8	360	XP_007990049
	CsKLF9	245	XP_007967675
	CsKLF10	481	XP_007999455
	CsKLF11	513	XP_007969699

	CsKLF12	403	XP_007958765
	CsKLF13	289	XP_008015385
	CsKLF14	324	XP_007981140
	CsKLF15	417	XP_007983710
	CsKLF16	253	XP_007992853
	CsKLF17	415	XP_007977211
**Rattus norvegicus (rat)**			
	RnKLF1	382	XP_006255316
	RnKLF2	352	NP_001007685
	RnKLF3	345	NP_001099212
	RnKLF4	483	NP_446165
	RnKLF5	444	NP_445846
	RnKLF6	318	NP_113830
	RnKLF7	95	NP_001102270
	RnKLF8	356	XP_008771325
	RnKLF9	245	NP_476559
	RnKLF10	481	NP_112397
	RnKLF11	502	NP_001032431
	RnKLF12	420	NP_001100751
	RnKLF13	132	NP_001102617
	RnKLF14	322	NP_001128566
	RnKLF15	416	NP_445988
	RnKLF16	252	NP_001121076
	RnKLF17	353	XP_008762309
